# The value of dynamic cerebral compliance monitoring after pediatric traumatic brain injury: a STARSHIP study sub-analysis

**DOI:** 10.1186/s13054-025-05403-w

**Published:** 2025-06-02

**Authors:** Stefan Yu Bögli, Ihsane Olakorede, Claudia Ann Smith, Marek Czosnyka, Peter Hutchinson, Shruti Agrawal, Peter Smielewski, the STARSHIP study team, Shruti Agrawal, Peter Smielewski, Peter J. Hutchinson, Stefan Yu Bögli, Claudia A. Smith, Carly Tooke, Caroline Payne, Holly Belfield, Amisha Mistry, Collette Spencer, Claire Jennings, Lara Bunni, Laura Anderson, Emily Morgan, Melanie James, Rebecca Beckley, Tahnima Khatun, Hafiza Khatun, Olivia Nugent, Richard Aldridge, Ruth Morgan, Julie Morcombe, Martin Quinton, Catherine Postlethwaite, Jenny Pond, Jessica Cutler, Caitlin Oxford, Marek Czosnyka, Michal Placek, Manuel Cabaleira, Deborah White, Esther Daubney, Adam Young, Erta Beqiri, Riaz Kayani, Roddy O’DonneII, Nazima Pathan, Suzanna Watson, Anna Maw, Matthew Garnett, Hari Krishnan Kanthimathinathan, Harish Bangalore, Santosh Sundararajan, Gayathri Subramanian, Dusan Raffaj, Simona Lampariello, Avishay Sarfatti, Anton Mayer, Oliver Ross

**Affiliations:** 1 Brain Physics Laboratory, Division of Neurosurgery, Department of Clinical Neurosciences, University of Cambridge, Cambridge, UK; 2 Division of Neurosurgery, Department of Clinical Neurosciences, University of Cambridge, Cambridge, UK; 3 Department of Paediatrics, Cambridge University, Cambridge, UK; 4 Paediatric Intensive Care, Cambridge University Hospitals, Cambridge, UK; 5 Institute of Electronic Systems, Warsaw University of Technology, Warsaw, Poland

**Keywords:** Traumatic brain injury, Compliance, Multimodality monitoring, Osmotherapy, Treatment

## Abstract

**Introduction:**

Cerebral compliance describes the pressure–volume relationship within the intracranial space, quantifying the brain’s capacity to accommodate changes in volume before significant increases in intracranial pressure (ICP) occur. The pulse shape index – PSI—classifies the ICP pulse-wave-configuration into 4 categories representing the incremental state of compliance. In this analysis we explore the metric in a cohort of prospectively collected pediatric TBI patients in relation to outcome, physiological parameters, and individual ICP insults.

**Methodology:**

Data acquired by the prospective observational STARSHIP study which included clinical information, 12-month outcome, and monitoring data from 98 pediatric TBI patients admitted to 10 pediatric intensive care units across the UK was assessed. PSI was calculated and compared using univariable and multivariable analyses, as well as considering their time-trends and relation to individual ICP insults.

**Results:**

PSI derived metrics were associated with outcomes within univariable analyses, additionally they were associated with ICP, and worse cerebrovascular reactivity (absolute correlation coefficients close to 0.3 for the described metrics). Cross correlation analysis revealed a median delay of 8 min for changes in ICP after changes in PSI (95% confidence interval of 7.6 to 8.5 min). Higher PSI value before ICP elevations was associated with longer duration and greater magnitude of subsequent ICP insults. Additionally, higher PSI before and faster decrease in PSI after osmotherapy were associated with successful reduction of ICP.

**Conclusions:**

PSI enhances bedside monitoring of pediatric TBI by enabling dynamic assessment of cerebral compliance. Variations in PSI appear to precede subsequent ICP insults and are associated with their severity, thereby potentially facilitating timely interventions. Furthermore, assessing the PSI level before administering osmotherapy may allow gauging treatment success.

**Supplementary Information:**

The online version contains supplementary material available at 10.1186/s13054-025-05403-w.

## Introduction

Cerebral compliance describes the pressure–volume relationship within the intracranial space, effectively quantifying the brain's capacity to accommodate changes in volume before significant increases in intracranial pressure (ICP) occur. High compliance allows for volume compensation with minimal ICP elevation, whereas low compliance results in marked pressure increases even in response to small volume changes. The capacity of cerebral compliance to buffer changes in volume is critical after traumatic brain injury (TBI) which usually brings about a variety of sequelae associated with brain tissue, blood or cerebrospinal fluid volume increases. Continuous monitoring of cerebral compliance can add to the absolute ICP value by providing insight into the brain’s ability to buffer increases in volume due to disease evolution or interventions.

Traditionally, the assessment of cerebral compliance necessitates modifying intracerebral volume either via the injection of fluid or adjustment of the volume of an intraventricular balloon (i.e. the Spiegelberg air-pouch ICP/compliance monitor). [1, 2] However, with continuous multimodality monitoring, further methods have been proposed, which do not necessitate the use of additional specific monitors or the infusion of fluid. Most recently, the pulse shape index (PSI) [3] has been developed based on the known effect of changes in pulse-wave morphology [4–8] attributed to decreasing compliance. PSI classifies the ICP pulse-wave shape into 4 categories (with one additional category representing the artefacts) which differentiate the waveforms into normal, possibly pathological, likely pathological and pathological respectively. Though PSI has been investigated to some extent in adults and has shown promise due to its association to outcome, ICP, and volume of space-occupying lesions [9–11], it has not been explored in children so far.

There are relevant physiological differences which preclude the direct adoption of results derived from adults to the pediatric TBI population. Most prominently, both the skull as well as the brain compartments (blood vs. brain vs. cerebrospinal fluid volume) go through age-related changes [12]. At birth, open fontanels and unfused sutures allow for some buffering of increases in ICP. Additionally, the ratio between brain tissue and CSF changes with an initial increase until the age of 7 and a secondary decrease thereafter [13).

Given the scarcity of available data and the unique factors that make direct inference from adult populations unfeasible, we aimed to provide an in-depth analysis of the pulse shape metric PSI in a prospective, multi-center cohort of pediatric TBI patients enrolled in the STARSHIP study [14].

## Materials and methods

The STARSHIP study was approved by the Health Research Authority, Southwest-Central Bristol Research Ethics Committee (Ref: 18/SW/0053 and 23/SW/0011). As the acute phase of the project was purely observational, a deferred consent was taken for acute data collection and sharing to allow for the acquisition of a bias-free sample. Informed consent was received by the patients’ legal guardian for data sharing and follow-up before hospital discharge.

### Study population

The patient data included in this study was acquired as part of the multi-center observational STARSHIP study (trial registration number: NCT05688462) including a total of 135 pediatric (age ≤ 16 years) TBI patients admitted to pediatric intensive care units in the United Kingdom (UK) between 2018 and 2023. All patients with pediatric TBI requiring invasive monitoring of arterial blood pressure (ABP) and ICP were eligible for recruitment; there were no specific exclusion criteria. The specifics of this trial were previously described in the published study protocol [15]. Patients who received a decompressive craniectomy or were below 12 months of age were excluded from this analysis since it remains unproven whether PSI is valid in patients without closed skull leading to a final cohort size of 98.

### Data acquisition

High resolution physiological data (250 Hz) was collected using the ICM + software (ICM + software ®, Cambridge Enterprises, University of Cambridge, UK). ICP was measured in the majority using intraparenchymal wires (Codman ICP MicroSensor, Codman & Shurtleff, Raynham, Massachusetts) and ABP was measured using arterial lines (Baxter Healthcare, Deerfield, Illinois) inserted in the radial or femoral artery zeroed at the level of the right atrium. The following clinical data was assessed: age, sex, type (isolated vs. polytrauma) and mechanism of TBI, initial Glasgow Coma Scale (GCS), pupillary reactivity (divided into both reactive, one reactive, none reactive), the injury severity score (ISS), the abbreviated injury scale (AIS) head, the Rotterdam CT score, incidence of pre-hospital episodes of hypotension or hypoxia, initial glucose and hemoglobin values, and the Glasgow Outcome Scale-Extended Pediatric Version (GOSE-Peds). GCS and pupillary reactivity were assessed after initial resuscitation, while the laboratory values and TBI severity descriptors were assessed at hospital admission. The additional timepoints were extracted: Time to Admission (time in hours between injury and admission to the intensive care unit) and Time to Monitoring (time in hours between injury and start of invasive monitoring). GOSE Peds was assessed at 12 months after ictus during outpatient consultations or via telephone interviews by trained staff. Outcome was evaluated either as unfavorable vs. favorable (GOSE Peds 5–8 vs. 1–4) or non-survivor vs. survivor (GOSE Peds 8 vs. 1–7).

### Data preprocessing

The high-resolution (i.e. waveform) monitoring was pre-processed using ICM + as previously described [14]. In essence, raw ABP signals were curated to remove sections with arterial line failure or sections outside of physiological ranges (i.e. below 0 or above 300 mmHg or sections with a pulse amplitude below 15 mmHg) while raw ICP traces were curated to remove sections outside of physiological ranges (i.e. ICP below −10 or above 200 mmHg) or without sufficient amplitude (i.e. < 0.04 mmHg) or with a 95% Spectral edge frequency above 10 Hz (high-frequency noise). The ICP amplitude was estimated as the fundamental (dominant peak) amplitude of ICP in frequency decomposition, within the limits of 40 to 180 Hz. The following metrics were calculated as previously described and assessed using minute-by-minute trends: PRx [16] – correlation coefficient between 10 s averages of ABP and ICP calculated over 5 min, optimal cerebral perfusion pressure (CPPopt [17] – i.e. CPP at the lowest PRx value identified by a parabolic curve fitted to 5 min median CPP and PRx values, with bins of CPP of 5 mmHg), and RAP [18] (index of cerebrospinal compensatory reserve, correlation coefficient between 10 s averages of ICP and ICP amplitude calculated over 5 min).

### The pulse shape index

**Fig. 1:**
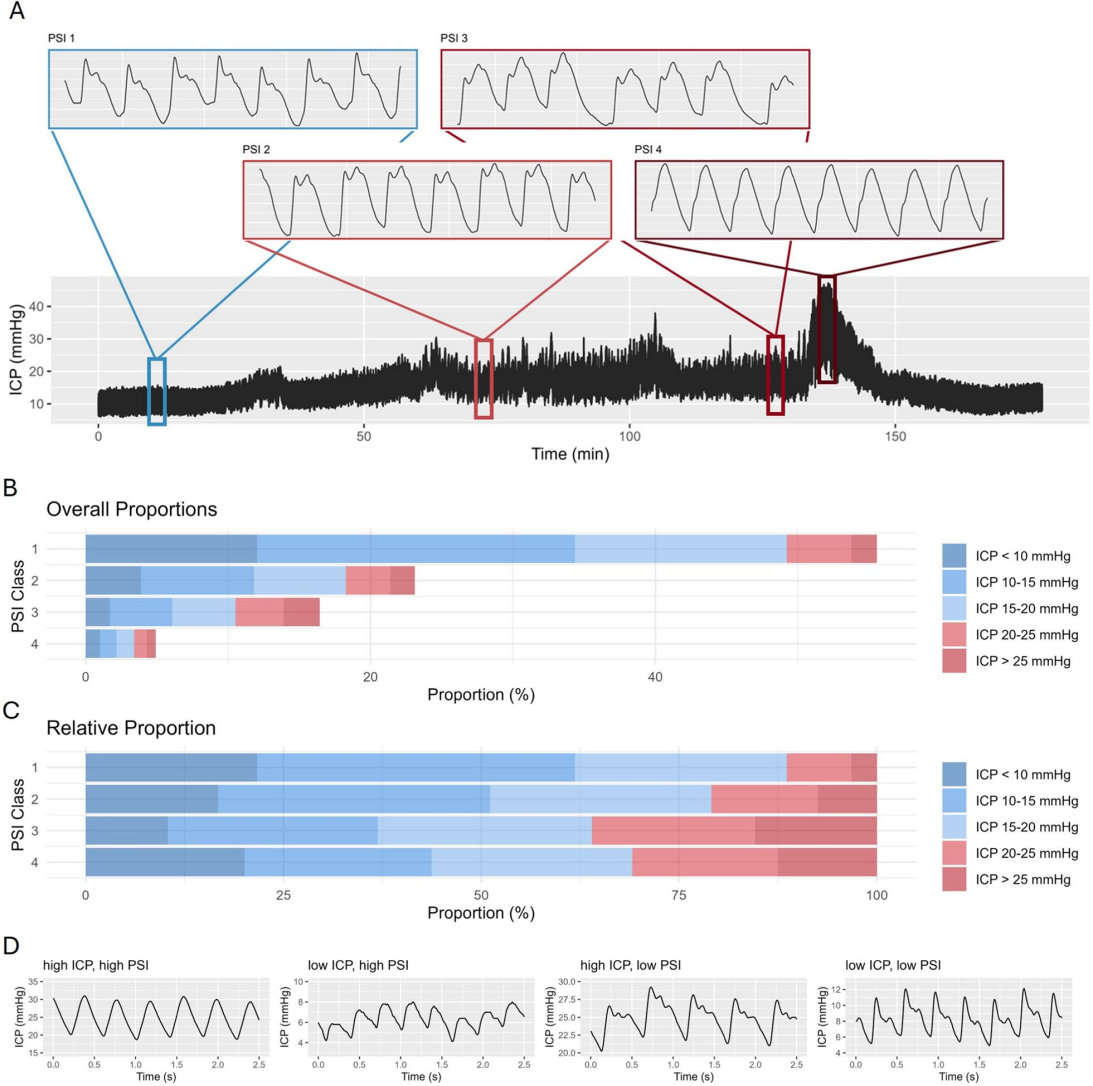
Intracranial pressure and pulse shape: Panel** A** presents a 3-h ICP time trend, with magnified PSI waveforms displayed above, illustrating the different PSI classes. The different classes describe the different ICP pulse waveform shapes (based on the relative relationships between the three peaks P1, P2, P3), which are related to different cerebral compliance states. The PSI index [3, 10] represents 5-min averages of classified waveforms within that time bin. Panel** B** and** C** display the overall and relative frequencies of different levels of ICP and the corresponding pulse shapes considering all patients. Panel** D** illustrates example sections where ICP and PSI are both high, both low, or diverging from each other

The pulse shape index – PSI—was calculated within the ICM + software using the model developed by Mataczynski et al.[3]. In essence, they developed a deep neural network model for the automated classification of ICP pulse waveforms, trained on data from adult TBI patients annotated manually by experts. The specific waveform categories identified by PSI are illustrated alongside a sample ICP trace in Fig. 1A. Based on the relationship between the three peaks in the ICP waveform (percussion wave P1, tidal wave P2, dicrotic wave P3) 4 compliance states were defined: 1 – dominant P1 (normal); 2 – accentuated P2, but P1 higher than P3 (potentially pathological); 3 – accentuated P2 and P3 (likely pathological); 4 – no visible peak/triangular shape (pathological). In keeping with definitions of other indices in ICM + PSI was configured to be calculated in moving 5-min segments with 80% overlap. Within the segment each and every pulse was classified, and the resulting class values were then averaged over the 5-min window resulting in a PSI numerical (floating point) value ranging between 1 and 4, allowing for short term variations in pulse shape. For further, statistical analysis PSI values were either evaluated as overall averages, per patient, or classified into normal (PSI below 2), high (above 2, but below 3), or critical (above 3). For each cutoff, we analyzed two measures: percentage time spent above the threshold (ptime) and dose, which represents the total burden of exposure. Dose was calculated as the area under the curve above the cutoff, considering the entire monitoring period and then normalized to the number of hours. This means that dose accounts for both how long and how far the values exceed the threshold.

### Statistical analysis

Statistical analysis and figure preparation was performed in R Studio (R version 4.4.1 – https://www.r-project.org/—packages used: *rstatix, gtsummary, MASS, ggplot2, corrplot*). Descriptive variables are reported as counts (percentages) or median (interquartile range – IQR). Distribution of the different continuous variables was assessed using the Shapiro–Wilk test. Equality of variances was tested using the Bartlett test or the Levene test. Different statistical methods were explored to assess the association between compliance metrics and outcome. Both univariable as well as multivariable analysis were performed.

### Outcome analysis

Clinical and multimodal monitoring metrics were assessed using Fisher’s exact test, Chi^2^ tests, or Wilcoxon rank sum tests as appropriate comparing either outcome (GOSE Peds 5–8 vs. 1–4) or mortality (GOSE Peds 8 vs. 1–7) as endpoint. Considering the relatively small number of patients with relevant heterogeneity in presentation we employed the following additional multivariable methods: Sliding dichotomy—The sliding dichotomy approach creates a relative outcome scale for each patient to account for differences in baseline parameters and clinical prognostic variables [19]. For this purpose, prognostic risk scores were estimated (using logistic regression comparing favorable vs. unfavorable outcome) for all patients taking various clinical parameters into account (Age, GCS motor, pupillary reactivity, ISS, Rotterdam Score, Hypoxia, Hypotension, Cardiac arrest). Based on these, the patients were then divided into three groups of roughly equal size with low, intermediate, and high likelihood of unfavorable outcome respectively. The definition of favorable and unfavorable outcome was then adjusted for each patient. Specifically, for patients with a low likelihood of unfavorable outcome only GOSE Peds ≤ 2 was considered favorable, while for the patients with intermediate likelihood GOSE Peds ≤ 4 and for the patients with high likelihood GOSE Peds ≤ 6 was considered favorable. The patients were then assessed using logistic regression based on this adjusted outcome definition. Second, considering the moderate sample size with many clinical and monitoring parameters available, multivariable models were built including the same clinical and additional monitoring parameters (ICP, CPP, PRx) and lastly the PSI measures, which were then fed through a backwards stepwise elimination process with a p-value threshold of 0.05 evaluated using the Akaike Information Criterion for comparison of the different models to allow for automated simplification of the models retaining only key prognostic parameters.

To allow for deeper insight into the time-dependence of the PSI outcome association, we explored visualizations based on the initial description by Guiza [20]. For this purpose, a publicly available code [21] was used and adapted to fit the current analysis. First, to explore the association between magnitude and duration of PSI changes and outcome, minute-by-minute PSI data was used to create a grid per patient describing the frequencies of different combinations of minimum intensity (range 1 to 4, 0.1 per cell) and minimum duration (range 5 to 120 min, 2 min per cell). To create the heatmaps, for each cell, weighted Pearson correlation (using the number of patients as weights) was used to assess the relationship between the average number of occurrences per outcome category. Second, to explore the impact of different combinations of ICP and PSI levels, the minute-by-minute PSI and ICP data was explored to create a grid per patient describing the percentage monitoring time with specific combinations of PSI (range 1 to 4, 0.1 per cell) and ICP (from 10 to 40 mmHg, 2 mmHg per cell). To create the heatmaps, for each cell, weighted Pearson correlation (using the number of patients as weights) was used to assess the relationship between the average percentage monitoring time and outcome category. The resulting maps were then visualized using a color map ranging from −1 (red) to 1 (blue) representing association to worse and better outcome respectively. Similarly, to assess statistical significance, correlation significance was assessed using t-statistics, with p-values adjusted via Bonferroni correction. A heatmap was generated, highlighting statistically significant correlations in blue. To improve visual interpretability, for all heatmaps, cells were smoothed as previously described [22], where each cell of the grid was divided into 3 * 3 smaller cells and this was followed by application of a Gaussian kernel filter (standard deviation of 2 pixels). Grid cells with less than 20 patients with the specific cell combination were colored gray.

### Clinical parameters and time trend analysis

Pearson and Spearman correlation coefficients were estimated to assess the strength and direction of association between PSI and clinical or multimodality monitoring metrics. Pearson correlation was used for continuous variables and Spearman correlation for ordinal metrics. Statistical significance of the correlations was assessed using t-statistics and visualized. To assess temporal aspects, we investigated the temporal relationships between PSI and ICP through two distinct analytical approaches: Cross-Correlation and Granger Causality analysis. First, Cross-Correlation was utilized to evaluate the temporal alignment between the two time-series across a range of time lags (in our case ± 15 min) for consecutive non-overlapping 1 h data segments, aiming to determine the optimal lag that maximizes the correlation. The lag was estimated considering only those sections for which the correlation coefficient reached 0.3 or higher. The resulting time lags are reported using the median with interquartile range (IQR), and a bootstrap-derived 95% confidence interval. Subsequently, Granger Causality calculations were conducted, within the same consecutive 1 h data segments, to ascertain whether the inclusion of past values of PSI could enhance the predictability of the ICP time series beyond its own historical values, thereby suggesting causal influence of PSI on ICP. For analysis, both optimal lag as well as magnitude of Granger Causality (quantified using the F-statistic) were averaged per patient.

### Event related analysis

Lastly, we assessed the relationship between PSI and individual ICP changes. First, we assessed the association between the state of PSI and subsequent ICP insults. For this purpose, we identified ICP insults, defined as episodes with ICP exceeding 20 mmHg for at least 5 min with a minimum ICP increase of 5 mmHg comparing before vs. during the insult. For analysis, physiological parameters were extracted from the 10-min segment before or the insult itself. The characteristics of the ICP insults were then compared to the state of PSI before the insult. Specifically, mixed effects models were employed to assess insults characteristics dependent on the value of PSI and ICP before the insult and adding the patient ID as a random effect to allow for a patient specific offset. Second, we investigated the effect of osmotherapy (i.e. hypertonic saline [23]) on PSI. The administration of osmotherapy was prospectively recorded at the bedside by nurses to ensure accurate timing. Our objective was to determine whether PSI could predict treatment success when osmotherapy was used to manage elevated ICP. To do this, we analyzed episodes of increased ICP, defined as ICP ≥ 20 mmHg for at least 5 min, that were subsequently treated with osmotherapy. Treatment success was defined as achieving either a sustained ICP below 20 mmHg for 30 min or a sustained ICP reduction of at least 10 mmHg for 30 min following osmotherapy. To examine the relationship between treatment success and PSI, we analyzed PSI trends before and after osmotherapy administration. Specifically, we assessed PSI changes during the 10 min preceding osmotherapy and across consecutive 5-min intervals up to 30 min post-administration, stratified by treatment success. To quantify differences in PSI trajectory, we applied mixed-effects models, using PSI time trends as the dependent variable, treatment success and timepoint as fixed effects (specifically assessing the interaction between these two variables), and patient ID as a random effect to account for inter-individual variability in physiology. Additionally, we examined whether PSI before osmotherapy could predict treatment success by applying a second mixed-effects model [24], where treatment success was the dependent variable, PSI before osmotherapy was the fixed effect, and patient ID was included as a random effect.

## Results

### Patients and monitoring characteristics

The patient cohort has previously been described in the primary analysis of the STARSHIP trial (Table 1). From a total of 124 patients with available 12-month outcome, 26 patients who were either below the age of 12 months or received a decompressive craniectomy were excluded leaving a final cohort of 98 patients (77% were male, median age 137 months). The clinical parameters are described in Table 1. Of the patients included, 45% suffered an isolated TBI with a median Rotterdam score of 3 (IQR 2, 3), an overall high trauma severity corresponding to a median ISS of 29 (IQR 25, 45) and an initial median GCS of 6 (IQR 4, 9). Overall availability of monitoring data is described in Supplement A. The median time from injury until start of invasive monitoring was 13 (IQR 6, 19) hours. Median monitoring metrics were: ICP: 14.3 (IQR 11.6, 16.7) mmHg; CPP: 65 (IQR 60, 69) mmHg, and PRx: −0.1 (IQR −0.2, 0.05). Specific differences by outcome are described in Supplement B and largely follow the results from the initial description. The median PSI within this patient cohort was 1.46 (IQR 1.15, 2.00). An example trace of ICP changes and corresponding PSI values is shown in Fig. 1A. Figure 1B and C display the frequency of different combinations between specific levels of ICP and PSI. Example sections displaying different combinations between high or low ICP and high or low PSI are shown in Fig. 1D.

**Table 1:** Patient characteristics

Characteristic	N = 98
Age (months)	137 (76, 158)
Sex (male)	75 (77%)
GCS	6 (4, 9)
GCS Motor	3 (1, 5)
*Pupillary Reactivity*	
both reactive	79 (85%)
one reactive	7 (7.5%)
none reactive	7 (7.5%)
ISS	29 (25, 45)
AIS Head	5 (4, 5)
Isolated TBI	44 (45%)
Rotterdam Score	3 (2, 3)
Hypoxia	10 (10%)
Hypotension	19 (20%)
Glucose (mmol/L)	6.20 (5.00, 7.19)
Hemoglobin (g/L)	112 (96, 125)
*Timepoints*	
Time to Admission (hours)	5.5 (3.5, 7.9)
Time to Monitoring (hours)	13 (6, 19)

The patient characteristics are described considering the whole cohort included with GCS and pupillary reactivity being assessed after initial resuscitation, and the laboratory values and TBI severity descriptors being assessed at hospital admission. The additional timepoints described represent the following information Time to Admission (time in hours between injury and admission to the intensive care unit); Time to Monitoring (time in hours between injury and start of invasive monitoring)

Data shown as Median (interquartile range) or number (%). Abbreviations: AIS—abbreviated injury scale; GCS—Glasgow Coma Scale; ISS—injury severity score; TBI—traumatic brain injury

### Outcome analysis

On a univariable basis, only few differences could be found (Table 2). Specifically, no difference could be found when comparing average PSI values depending on outcome (median 1.44 (IQR 1.21, 1.93) for favorable versus 1.56 (IQR 1.05, 2.17) for unfavorable respectively, *p* = 0.9) or depending on mortality (median 1.46 (IQR 1.15, 1.97) for survivors versus 2.30 (IQR 2.30, 2.64) for non-survivors respectively, *p* = 0.093). However, critically increased PSI dose was different depending on the outcome (median 6 (IQR 0, 130) for favorable versus 41 (IQR 5, 257) for unfavorable respectively, *p* = 0.025) and mortality (median 7 (IQR 0, 148) for survivors versus 85 (IQR 80, 337) for non-survivors respectively, *p* = 0.045). Additionally, percentage time spent with critically increased PSI was higher in non-survivors (median 1% (IQR 0, 4) for survivors versus 4% (IQR 3, 17) for non-survivors respectively, *p* = 0.048).

**Table 2:** Clinical Metrics stratified by mortality or outcome

Outcome			
Characteristic	Favorable N = 69	Unfavorable N = 29	*p*-value
PSI (mean)	1.44 (1.21, 1.93)	1.56 (1.05, 2.17)	0.9
PSI dose high	343 (57, 1,348)	659 (43, 3,018)	0.5
PSI dose critical	6 (0, 130)	41 (5, 257)	0.025
PSI ptime high (%)	14 (3, 42)	19 (1, 60)	0.8
PSI ptime critical (%)	0 (0, 4)	1 (0, 6)	0.11
PSI (while ICP below 20 mmHg; mean)	1.39 (1.16, 1.82)	1.34 (1.03, 2.03)	0.5
PSI (while ICP below 15 mmHg; mean)	1.33 (1.12, 1.68)	1.21 (1.02, 1.92)	0.4

Mortality			
Characteristic	Survivor N = 93	Non-survivor N = 5	*p*-value
PSI	1.46 (1.15, 1.97)	2.30 (2.30, 2.64)	0.093
PSI dose high	314 (43, 1,497)	2,802 (2,202, 3,255)	0.081
PSI dose critical	7 (0, 148)	85 (80, 337)	0.045
PSI ptime high	12 (2, 42)	71 (65, 87)	0.073
PSI ptime critical	1 (0, 4)	4 (3, 17)	0.048
PSI (while ICP below 20 mmHg)	1.39 (1.12, 1.85)	1.56 (1.05, 2.32)	> 0.9
PSI (while ICP below 15 mmHg)	1.29 (1.11, 1.71)	1.46 (1.01, 2.24)	0.7

The table describes the univariable analysis comparing the different PSI derived metrics stratified by outcome (favorable vs. unfavorable) or mortality (survivors vs. non-survivors). The PSI metrics assessed are overall mean PSI, PSI dose and ptime (i.e. area under the curve above the cutoff or percentage monitoring time spent above the cutoff respectively) for the PSI categories high (PSI between 2 and 3) and critical (PSI above 3), and mean PSI during sections with normal and low ICP (i.e. ICP below 20 and 15 mmHg respectively)

Data shown as Median (interquartile range). Abbreviations: critical – PSI above 3; high – PSI between 2 and 3; PSI – Pulse Shape Index; ptime – percentage time within either state; ICP – intracranial pressure

Two multivariable methods were explored for further exploration. First, we utilized a sliding dichotomy-based approach (Supplement C). No PSI metric differences could be found after adjusting the outcome definition based on the clinical presentation. Second, backwards stepwise regression including all patients and starting from a model including all the clinical and monitoring metrics and either one of the PSI descriptors showed no relevant additional benefit of compliance metrics for prognostication over and above the known clinical predictors.

**Fig. 2:**
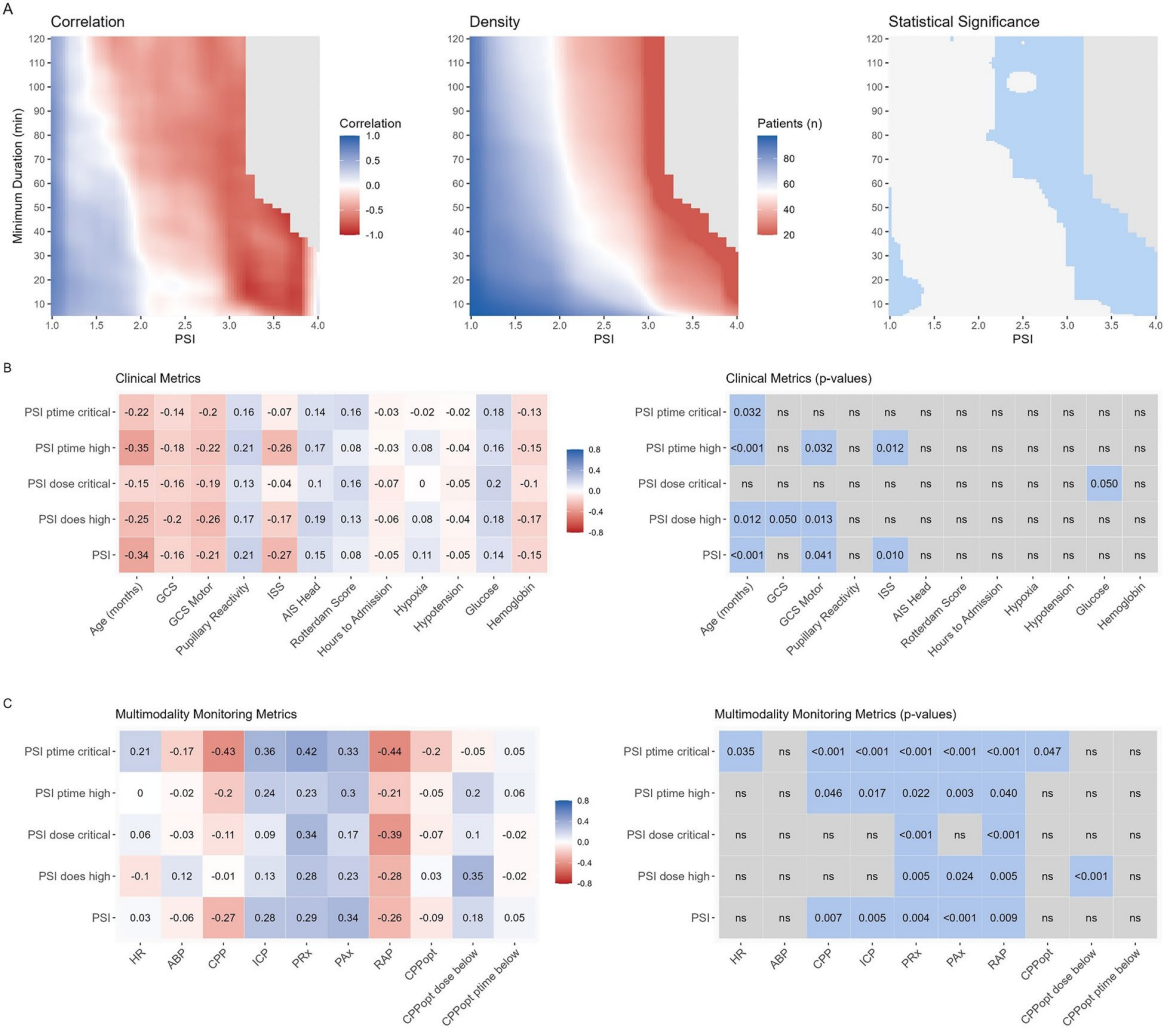
Pulse Shape Index Heatmap – time dependence and correlation to clinical and multimodality monitoring metrics. Panel** A** presents three heatmaps. The left heatmap illustrates the correlations between different PSI levels, their respective minimum durations, and patient outcomes. Red indicates an association with unfavorable outcomes, whereas blue indicates an association with favorable outcomes. Notably, a shift in association occurs around PSI values 1.5 to 2, depending on the duration spent at that state. The middle heatmap shows the number of patients included in each cell, with higher counts in blue and lower counts in red. The right heatmap displays the results of the statistical analysis, with significant cells highlighted in blue. For all heatmaps, cells containing fewer than 20 patients are shown in gray. Panels** B** and** C** each depict correlations on the left—between PSI and clinical metrics (Panel** B**) or between PSI and multimodality monitoring metrics (Panel** C**). Here, color indicates the magnitude and direction of correlation (blue for positive, red for negative). On the right side of both panels, the corresponding significant p-values are shown

Lastly, to explore whether there might be a time dependent aspect, heatmaps were built assessing the insult intensity against duration (Fig. 2A). A shift in outcome association was observed for PSI values between 1.5 and 2, depending on the duration of the insult. Furthermore, PSI values exceeding 3 showed a progressively stronger association with worse outcomes, with statistical significance reached above 3.5 and at lower PSI values for prolonged insult durations. Similarly, we explored whether the combination of increased PSI and ICP would be worse (Supplement D). No consistent patterns describing stronger associations between specific combinations of ICP, PSI, and outcome could be identified.

### Clinical parameters and time trend analysis

Considering that the compliance, as indicated by PSI index, describes the state of intracranial space rather than a type of injury itself, we explored its physiological origin using different methods. First, we explored the correlation between PSI and different clinical and monitoring parameters (Fig. 2 C and D). When exploring overall associations, there were weak associations to clinical parameters describing higher clinical severity (e.g. correlation of −0.27 between PSI and ISS and −0.21 between PSI and GCS motor; *p* = 0.01 and 0.041 respectively). Slightly stronger correlations were found between increasing age and lower PSI value (correlation coefficient −0.34 for PSI vs. age, *p* < 0.001). Along the same lines there were weak up to moderate correlations between worse compliance and brain biosignals (e.g. correlation coefficient −0.27 between PSI and CPP (*p* = 0.007), 0.28 between PSI and ICP (*p* = 0.005), and 0.29 between PSI and PRx (*p* = 0.004)). The strongest correlation was found between the percentage time spent with critically increased PSI and higher PRx (0.42, *p* < 0.001) and lower CPP (−0.43, *p* < 0.001).

Considering the weak point-by-point correlation between PSI and ICP, and the physiological property that PSI aims to describe (i.e. compliance), we were interested in exploring additional measures to see its value for continuous monitoring. Three additional analyses were performed for this purpose: Cross-correlation, Granger Causality and Insult-related analysis. First, we explored cross-correlation between PSI and ICP over a range of time lags from −15 to + 15 min. The analysis revealed a median positive lag of 8 min (IQR: 7–9), with a 95% confidence interval of 7.6 to 8.5 min, suggesting a delayed response in ICP following changes in PSI. Second, we applied Granger causality analysis, which indicated a median causality magnitude of 0.37 (IQR 0.31, 0.43, *p* < 0.05) suggesting a positive predictive influence of past PSI values on subsequent ICP readings.

### Event related analysis

**Fig. 3:**
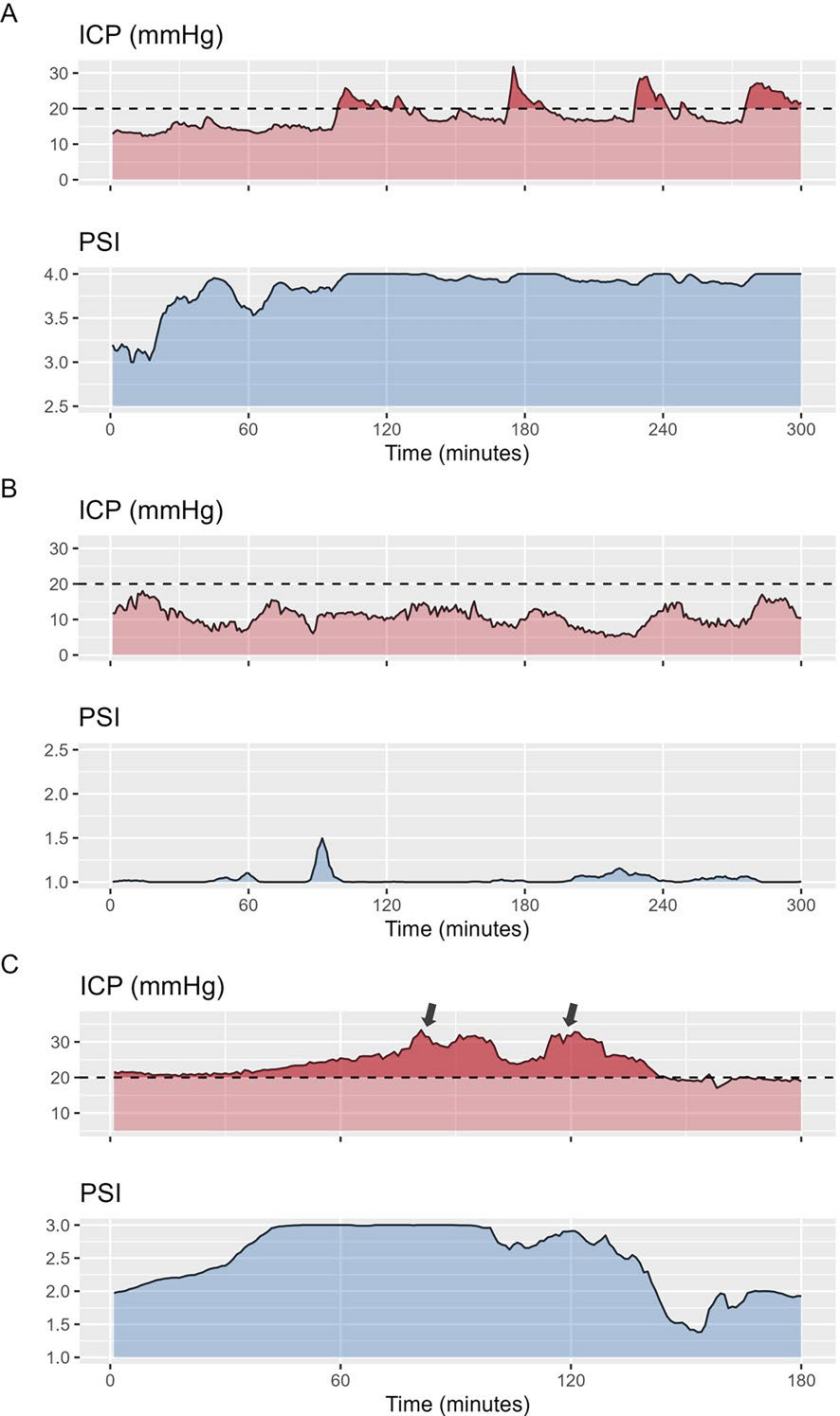
Example sections. Panel** A** and** B** display two 5-h example sections. Both start with intracranial pressure (ICP) around 10 to 15 mmHg. In case A the pulse shape index (PSI) is already high at the beginning and increases to values close to 4 without corresponding changes in ICP. This increase is then followed by reoccurring ICP insults lasting up to 60 min. In case B, a highly dynamic ICP trace can be appreciated without corresponding changes in PSI or ICP insults. In case **C**, a shorter section covering 3 h is shown, where a distinct rise in PSI can be appreciated within the first hour of the section with a delayed increase in ICP, ultimately treated with osmotherapy (black arrows). While the initial administration only leads to a short-lasting improvement in PSI and ICP, the second administration is associated with a distinct improvement of PSI decreasing from 3 to below 2 and a sustained decrease in ICP

Lastly, we explored the dynamics of PSI in relation to ICP insults. Example sections displaying the association between changes in PSI and ICP can be found in Fig. 3 A and B. For this purpose, PSI prior to the insults was differentiated into normal, high, and critical. Patients with critical increases in PSI before the insults suffered longer insults and higher ICP doses in the subsequent insults (Table 3). This association could also be shown within mixed effects models including average PSI and ICP before the insult as fixed effects and the Patient ID as a random effect (Table 3). Specifically, average PSI before the insults remained independently positively associated with ICP insult dose (Beta coefficient = 98 (CI: 12–184)) and highest ICP during the insult (Beta coefficient = 3.8 (CI: 0.79–6.7) irrespective of the ICP before the insult and the patient specific random effect.

**Table 3:** Insult characteristics dependent on prior compliance state

A—Univariable analysis	PSI category before insult			*p*-values		
Insults characteristic	Normal	High	Critical	Normal versus high	Normal versus critical	High versus critical
Mean ICP (mmHg)	25.2 (23.6, 29.0)	24.9 (23.1, 28.1)	25.3 (24.9, 26.8)	0.6	> 0.9	0.8
Max ICP (mmHg)	33 (29, 41)	36 (30, 45)	32 (29, 32)	0.2	0.4	0.3
ICP dose (mmHg)	86 (47, 203)	119 (67, 188)	199 (170, 455)	0.2	0.023	0.054
Insult duration (min)	14 (9, 26)	20 (11, 36)	55 (25, 63)	0.10	0.005	0.032

B—Mixed Effects Models	Fixed effect	Beta coefficient	Confidence interval
Model 1 – ICP dose (mmHg)	Mean PSI	98	12–184
	Mean ICP	20	−1.5–42
Model 2 – Max ICP (mmHg)	Mean PSI	3.8	0.79–6.7
	Mean ICP	0.68	−0.03–1.4

The univariable analysis of associations between the PSI category before the insult and the insult characteristics is shown in the top rows (A). The statistical analysis was performed via pairwise comparison of insult characteristics depending on the different PSI categories (normal (below 2), high (above 2, but below 3), or critical (above 3)). The specific insult characteristics assessed were mean and max ICP, ICP dose (i.e. area under the curve above the cutoff normalized to hourly values), and insult duration. The bottom rows display the results of the mixed effects models assessing the effect of average PSI before the insult on the insult characteristics when adding patient ID and insult number as random effects and ICP before the insult as fixed effect (B). The beta coefficients represent the extent of change in the response variable (i.e. ICP dose or max ICP) relative to a step increase in the input variable (i.e. PSI or ICP)

Data shown as Median (interquartile range). Abbreviations: PSI – Pulse Shape Index; ICP – intracranial pressure

**Fig. 4:**
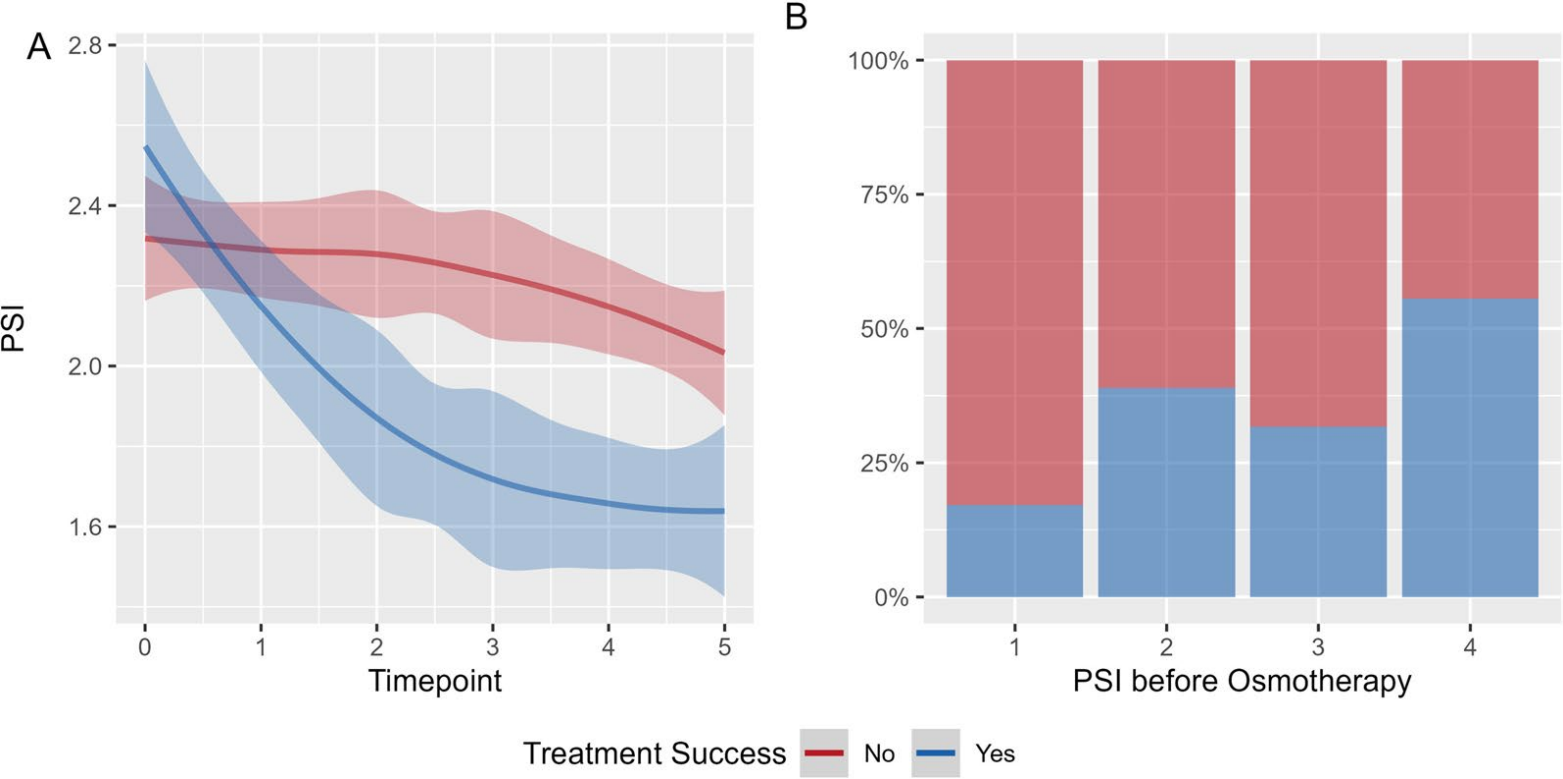
Pulse Shape Index and Treatment Response. Panel** A** illustrates the PSI levels over time, stratified by treatment response. Treatment response was defined as either sustained ICP below 20 mmHg for 30 min or a sustained ICP reduction of at least 10 mmHg for 30 min following osmotherapy. Timepoint 0 represents the 10 min preceding osmotherapy, while timepoints 1–5 correspond to consecutive non-overlapping 5-min intervals from 5 to 30 min after administration. For visualization, we applied a locally weighted scatterplot smoothing method, displaying the fitted curve along with its 95% confidence interval. Although PSI decreased in both groups, the reduction was more pronounced in patients where treatment successfully lowered ICP. Panel** B** depicts the proportion of patients achieving treatment response based on PSI levels before osmotherapy. Overall, a higher PSI prior to treatment was associated with an increased likelihood of treatment response

Lastly, we explored the predictive information of PSI for assessing treatment response. A total of 162 administrations of osmotherapy were identified with 51 successful and 111 unsuccessful administrations respectively. An example trace displaying PSI before and after a successful and one unsuccessful administration of osmotherapy can be appreciated in Fig. 3 C. Figure 4 panel A displays the PSI time trends depending on treatment response. When assessed within mixed effects models, there was a significant difference in trajectory dependent on treatment success with a steeper decrease in PSI in those with successful ICP reduction (*p* < 0.001). Figure 4 panel B displays the proportion of treatment response depending on PSI before osmotherapy. The proportion of treatment success were 0.17 for PSI 1, 0.38 and 0.32 for PSI 2 and 3, and 0.56 for PSI 4 respectively. When assessed within a mixed effects model, there was an increase in the likelihood of successful treatment (odds ratio 1.76 (CI 1.05–2.96; *p*-value = 0.033)) with higher PSI prior to osmotherapy.

## Discussion

ICP monitoring is a fundamental component of intensive care management for TBI. Traditionally, ICP has been characterized by its mean value, providing an important, albeit relatively crude metric to clinicians. Importantly, the utility of ICP extends beyond this simplistic measure. The ICP waveform configuration is often intuitively explored at the bedside and allows for inference of additional information [25]. Unlike the visual interpretation commonly performed by clinicians [26], the PSI enhances the value of ICP monitoring by providing a quantitative dynamic assessment of the pulse shape, believed to reflect the cerebral compliance. We explored this metric, for the first time, in a prospectively acquired pediatric cohort of TBI patients.

Although PSI-derived metrics were associated with outcome in univariable analyses, these associations did not remain significant after adjusting for relevant clinical prognostic markers. The lack of a strong association should not be surprising, given that an impairment of compliance does not inherently represent secondary brain injury. Rather, it is a risk factor for such injury because of the sensitivity of intracranial pressure dynamics to changes in volume. Far more significant than prognostication is the identification of potentially actionable physiological changes. Importantly, we found that changes in PSI (similar to findings in adult explorations of compliance metrics [27]) preceded changes in ICP and identified high PSI (indicative of impaired compliance) to be associated with worse subsequent ICP insults. Additionally, we identified higher PSI to be associated with higher likelihood of treatment success when considering the administration of osmotherapy for the management of intracranial hypertension. Currently, the evidence supporting hyperosmolar therapy in pediatric TBI remains limited [23], in part due to the small number of available studies—only some of which have demonstrated a benefit for managing ICP [28]. The found dependence on pulse shape (representing a surrogate measure of compliance) might shed some light on the lack of stronger evidence. Hyperosmolar therapy primarily works by creating an osmotic gradient between the blood and brain tissue [29]. After administration, by increasing serum osmolality, fluid is drawn out of the brain tissue into the vascular compartment [30]. This transfer of volume improves compliance [31] since unlike the brain tissue, the blood volume can be reduced to accommodate for elevated ICP through increased venous drainage. If compliance is relatively preserved in spite of increased ICP, osmotherapy, which increases the blood volume might not reduce ICP and even decrease compliance [32], for example in episodes associated with hyperemia [33].

The assessment of PSI reflects current bedside management practices, during which clinicians assess the brain’s compliance by examining instantaneous ICP pulse shapes on bedside monitors. Monitoring PSI continuously at the bedside [34] allows for visualizing dynamic trends that allow for ongoing, real-time assessments. The value of monitoring trends within individual patients, rather than relying on absolute values, might be particularly important in pediatric patient cohorts, as children’s skulls and intracranial compartments (blood, brain, and cerebrospinal fluid volumes) undergo age-related changes [12, 13]. Moreover, the pressure–volume curve differs across diseases, age groups, and individual patients [35, 36]. An additional appealing benefit of using PSI is that it relies neither on an additional probes or examination (e.g. transcranial doppler analyses [37[) nor on the active adjustment of intracerebral volume [1, 2] allowing for its exploration in any patient receiving invasive ICP monitoring.

### Limitations

Despite the multi-center design with inclusion of pediatric TBI patients across 10 centers within the UK, the resulting sample size is small – both due to the scarceness of pediatric TBI as well as since we excluded patients without intact skull (a cohort for which it is unclear whether PSI is valid). Consequently, no further subgrouping was possible to account for potential differences depending on age or type of injury. Furthermore, PSI was originally developed in adult patients with TBI and subarachnoid hemorrhage(3). While this may seem like a clear limitation, the variation in disease severity and hemorrhage extent among the included adults allowed for the assessment of various intracranial compartment changes that are similar to those observed in pediatric patients. Of note, research on pediatric TBI remains limited overall, and although distinct ICP waveforms are certainly detectable in pediatric patients, it is not yet clear whether waveform changes identified in adults can be interpreted in the same way in pediatric settings.

The rise of bedside available, real-time analysis tools like ICM + has enhanced the applicability of complex models, such as those used for estimating PSI, for clinical practice [34]. From a practical standpoint, PSI is readily implementable at the bedside, since the trained model is openly available on GitHub [3] and can be integrated into any monitoring platform that supports real-time signal analysis such as ICM + or the Moberg Cloud Platform. Future research should prioritize evaluating these metrics directly at the bedside, rather than relying on retrospective analyses, as was done in the present study. Of particular importance will be the assessment of different measures of therapeutic intensity (e.g. Pediatric Intensity Level of Therapy score [38]) and whether the found time lags and associations are of use within interventional studies. Additionally, it is worthwhile to mention that other technologies such as the Brain4 Care device [39, 40] are being developed allowing for non-invasive monitoring of intracranial dynamics (cerebral compliance indicator and through that also intracranial hypertension indicator). Such devices might ultimately allow for a fuller picture of cerebral compliance in a wider range of patients and diseases.

## Conclusions

The pulse shape index PSI provides an intuitive, interpretable method for continuous, automated quantification of the ICP waveform shape, and may prove beneficial for monitoring pediatric TBI by offering indirect insights into intracranial compliance. Increases in PSI precede increases in ICP suggesting a potential window for therapeutic intervention. Additionally, higher PSI before administration of osmotherapy, representative of decreased compliance, is associated with higher likelihood for successful ICP management.

## Supplementary Information

Supplementary materials 1: Supplement A-D

Supplementary materials 2: STARSHIP Study Team

## Data Availability

Postprocessed data is available upon reasonable request to the corresponding author.

## Abbreviations

ABP: Arterial blood pressure
AIS: Abbreviated injury scale
CPP: Cerebral perfusion pressure
CPPopt: Optimal CPP
GCS: Glasgow coma scale
GOSE Peds: Glasgow outcome scale-extended pediatric version
ICP: Intracranial pressure
IQR: Interquartile range
ISS: Injury severity score
PRx: Pressure reactivity index
PSI: Pulse shape index
RAP: Index of cerebrospinal compensatory reserve
TBI: Traumatic brain injury
