# Supplementary material for: The value of dynamic cerebral compliance monitoring after pediatric traumatic brain injury: a STARSHIP study sub-analysis
Source: Crit Care. 2025 Jun 2;29:219. doi: 10.1186/s13054-025-05403-w (PMC12128296; doi:10.1186/s13054-025-05403-w)
Supplement: Supplementary file 1 — Supplementary materials 1: Supplement A-D [file 13054_2025_5403_MOESM1_ESM.docx]

# SUPPLEMENT

**Supplement A. Data coverage.** The data coverage is shown in form of boxplots (length), histograms (frequency per length) and density (frequency of data depending on day post TBI with day 0 being the day of initial TBI). On average, over 4 days of high-resolution monitoring data was available for ABP, ICP, and their derivatives (A). The distribution of available data durations per patient was similar considering ABP and ICP (B). Within the density plot (C) a distinct peak in availability of data between day 1 and 4 after the initial TBI (day 0) can be seen. The median time between the initial TBI and admission to the PICU/start of multimodality monitoring was 13 hours.


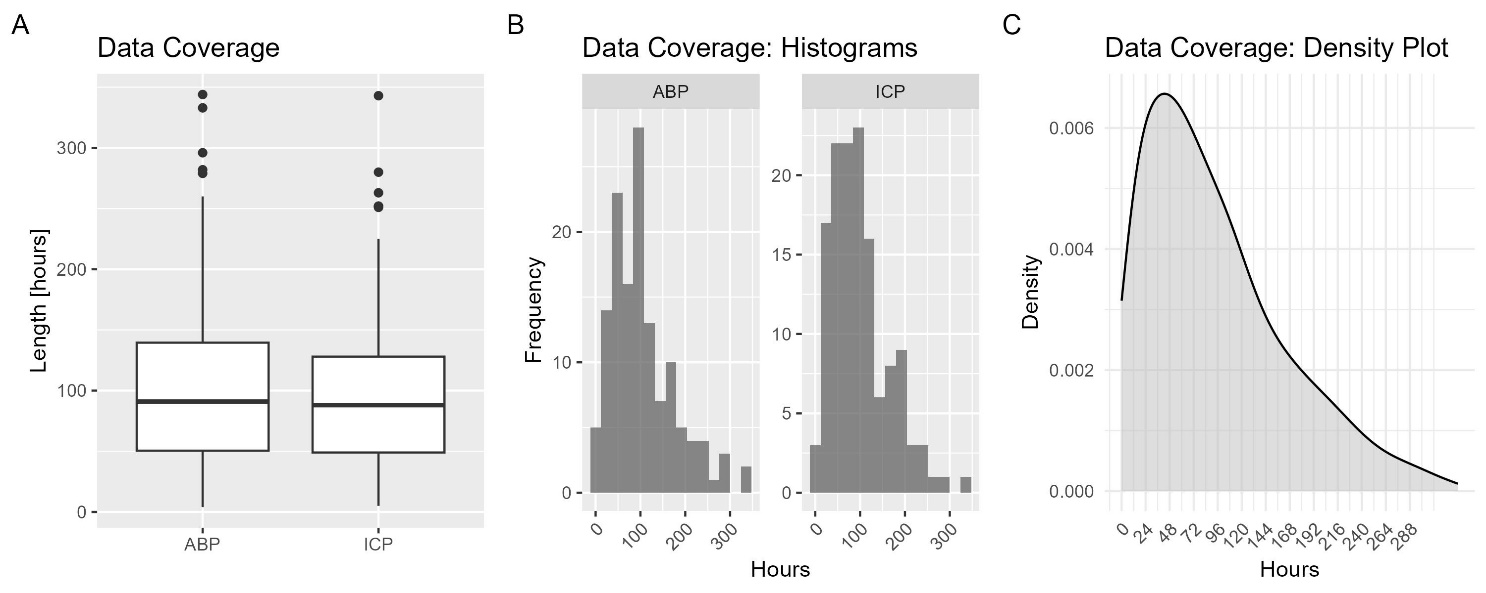


**Supplement B. Clinical and General Monitoring Characteristics by Outcome.**

**
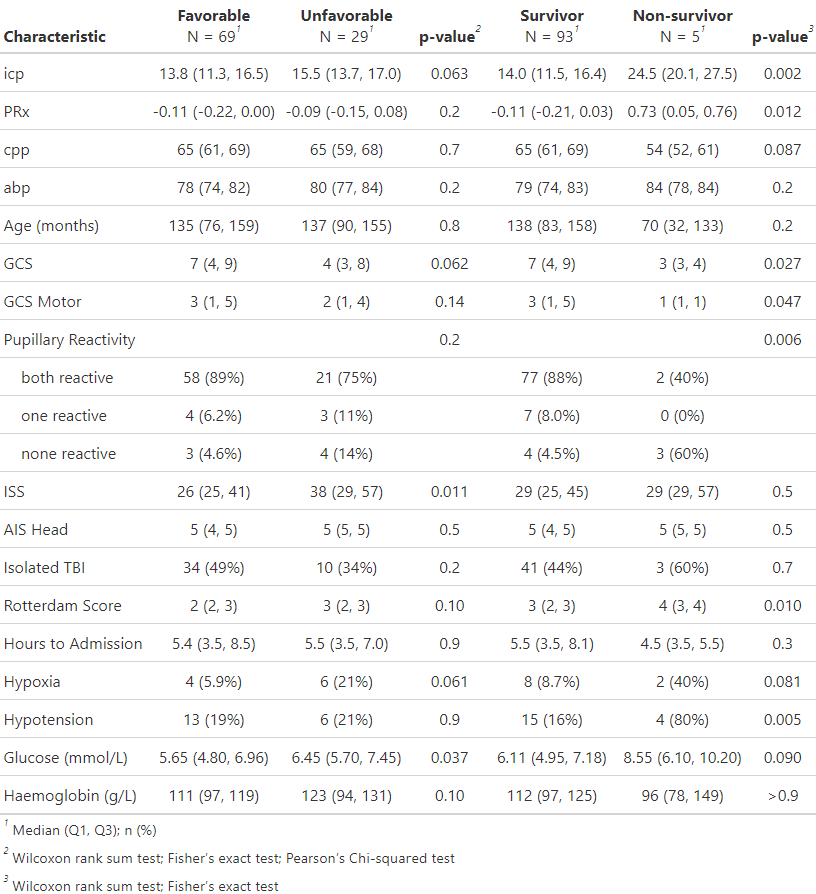
**

**Supplement C. Sliding Dichotomy Results.** The results of the logistic regression analyses relative to the adjusted outcome definition identified using the sliding dichotomy methods are shown. No significant associations could be identified.

| **Characteristic** | **OR (95% CI)** | **p-value** |
| --- | --- | --- |
| PSI | 1.16 (0.61-2.16) | 0.6 |
| PSI dose high‡ | 1.00 (0.98-1.02) | 0.9 |
| PSI dose critical‡ | 1.00 (0.95-1.05) | 0.9 |
| PSI ptime high† | 1.01 (0.94-1.07) | 0.8 |
| PSI ptime critical† | 1.07 (0.96-1.19) | 0.2 |
| PSI (while ICP below 20 mmHg) | 0.94 (0.46-1.84) | 0.9 |
| PSI (while ICP below 15 mmHg) | 0.98 (0.98, 1.93) | 0.9 |

* Abbreviations: critical – PSI above 3; high – PSI between 2 and 3; ptime – percentage time within either state; PSI – Pulse Shape Index; ICP – intracranial pressure;

‡per increase in dose of 100;

†per increase in ptime of 5%.


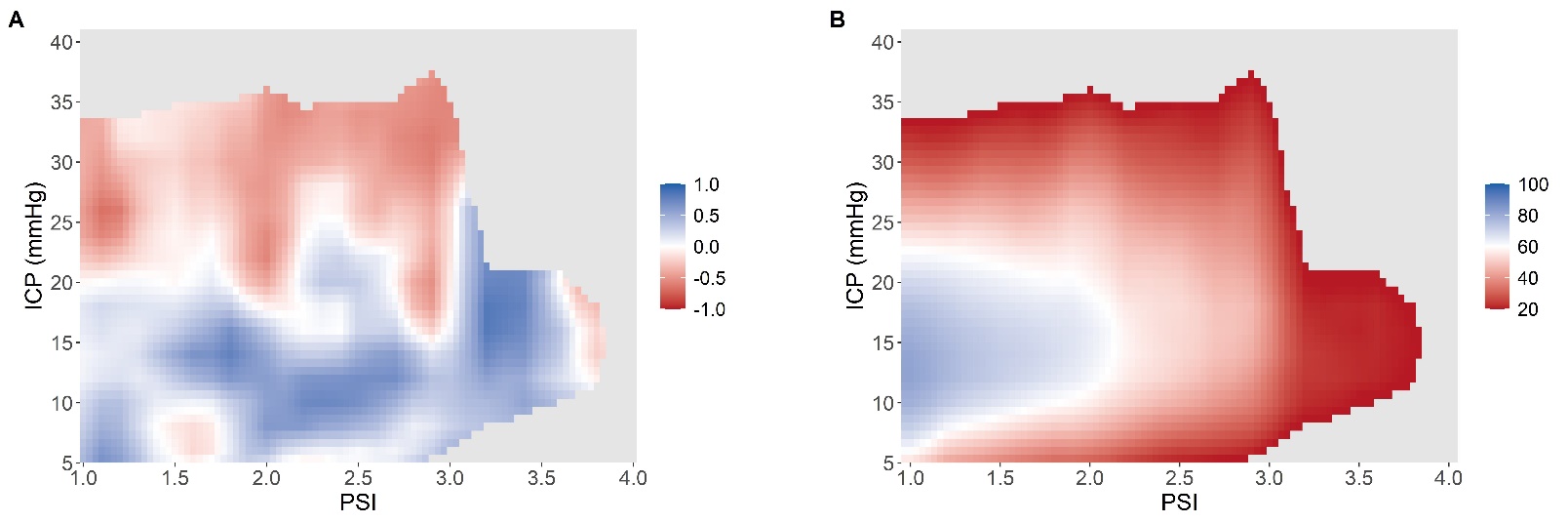
**Supplement D. Heatmaps PSI vs. ICP.** In panel A, specific combinations of different ICP and PSI levels are shown in relation to outcome with red representing association to unfavorable outcomes and blue associations to favorable outcomes. In panel B, the number of patients included within the respective cells are shown with blue representing higher and red representing lower number. Cells with less than 20 patients were colored gray. Overall, only inconsistent associations could be found.
