# Supplementary material for: The value of dynamic cerebral compliance monitoring after pediatric traumatic brain injury: a STARSHIP study sub-analysis
Source: Crit Care. 2025 Jun 2;29:219. doi: 10.1186/s13054-025-05403-w (PMC12128296; doi:10.1186/s13054-025-05403-w)
Supplement: Supplementary file 2 — Supplementary materials 2: STARSHIP Study Team [file 13054_2025_5403_MOESM2_ESM.pdf]

STARSHIP Study Team

First Name	Last Name	Institution	Location	Role
Shruti	Agrawal	Cambridge University Hospitals	Cambridge	Principal Investigator
Peter	Smielewski	University of Cambridge	Cambridge	Principal Investigator
Peter J.	Hutchinson	Cambridge University Hospitals	Cambridge	Principal Investigator
Stefan Yu	Bögli	University of Cambridge	Cambridge	Postdoc Researcher
Claudia A.	Smith	University of Cambridge	Cambridge	PhD Candidate
Carly	Tooke	Birmingham Children's Hospital	Birmingham	Research nurse
Caroline	Payne	Great Ormond Street Hospital	London	Research nurse
Holly	Belfield	Great Ormond Street Hospital	London	Research nurse
Amisha	Mistry	Leeds Children's Hospital	Leeds	Research nurse
Collette	Spencer	Leeds Children's Hospital	Leeds	Research nurse
Claire	Jennings	Royal Manchester Children's Hospital	Manchester	Research nurse
Lara	Bunni	Royal Manchester Children's Hospital	Manchester	Research fellow
Laura	Anderson	Nottingham Children's Hospital	Nottingham	Research nurse
Emily	Morgan	Nottingham Children's Hospital	Nottingham	Research nurse
Melanie	James	Oxford University Hospitals	Oxford	Research nurse
Rebecca	Beckley	Oxford University Hospitals	Oxford	Research nurse
Tahnima	Khatun	Royal London Hospital	London	Research nurse
Hafiza	Khatun	Royal London Hospital	London	Research nurse
Olivia	Nugent	Royal London Hospital	London	Research nurse
Richard	Aldridge	Royal London Hospital	London	PICU technician
Ruth	Morgan	Sheffield Children's Hospital	Sheffield	Research nurse
Julie	Morcombe	Sheffield Children's Hospital	Sheffield	Research nurse
Martin	Quinton	Sheffield Children's Hospital	Sheffield	PICU technician
Catherine	Postlethwaite	University Hospitals Southampton	Southampton	Research nurse
Jenny	Pond	University Hospitals Southampton	Southampton	Research nurse
Jessica	Cutler	University Hospitals Southampton	Southampton	Research nurse
Caitlin	Oxford	University Hospitals Southampton	Southampton	Research nurse
Marek	Czosnyka	University of Cambridge	Cambridge	Site investigator
Michal	Placek	University of Cambridge	Cambridge	Postdoctoral Researcher
Manuel	Cabaleira	University of Cambridge	Cambridge	Research Associate
Deborah	White	Cambridge University Hospitals	Cambridge	Research nurse
Esther	Daubney	Cambridge University Hospitals	Cambridge	Research nurse
Adam	Young	University of Cambridge	Cambridge	Site investigator
Erta	Beqiri	University of Cambridge	Cambridge	PhD Candidate
Riaz	Kayani	Cambridge University Hospitals	Cambridge	Site investigator
Roddy	O'Donnell	Cambridge University Hospitals	Cambridge	Site investigator
Nazima	Pathan	University of Cambridge	Cambridge	Site investigator
Suzanna	Watson	Cambridge Centre for Paediatric Neuropsychological Rehabilitation	Cambridge	Paediatric Neuropsychologist
Anna	Maw	Cambridge University Hospitals	Cambridge	Site investigator
Matthew	Garnett	Cambridge University Hospitals	Cambridge	Site investigator
Hari Krishnan	Kanthimathinathan	Birmingham Children's Hospital	Birmingham	Site investigator
Harish	Bangalore	Great Ormond Street Hospital	London	Site investigator
Santosh	Sundararajan	Leeds Children's Hospital	Leeds	Site investigator
Gayathri	Subramanian	Royal Manchester Children's Hospital	Manchester	Site investigator
Dusan	Raffaj	Nottingham Children's Hospital	Nottingham	Site investigator
Simona	Lampariello	Oxford University Hospitals	Oxford	Site investigator
Avishay	Sarfatti	Royal London Hospital	London	Site investigator
Anton	Mayer	Sheffield Children's Hospital	Sheffield	Site investigator
Oliver	Ross	Southampton General Hospital	Southampton	Site investigator
